# Transcriptome signature for dampened Th2 dominance in acellular pertussis vaccine-induced CD4^+^ T cell responses through TLR4 ligation

**DOI:** 10.1038/srep25064

**Published:** 2016-04-27

**Authors:** Jolanda Brummelman, René H. M. Raeven, Kina Helm, Jeroen L. A. Pennings, Bernard Metz, Willem van Eden, Cécile A. C. M. van Els, Wanda G. H. Han

**Affiliations:** 1 Centre for Infectious Disease Control, National Institute for Public Health and the Environment, Bilthoven, The Netherlands; 2 Department of Infectious Diseases and Immunology, Utrecht University, The Netherlands; 3 Institute for Translational Vaccinology (Intravacc), Bilthoven, The Netherlands; 4 Centre for Health Protection, National Institute for Public Health and the Environment, Bilthoven, The Netherlands

## Abstract

Current acellular pertussis (aP) vaccines promote a T helper 2 (Th2)-dominated response, while Th1/Th17 cells are protective. As our previous study showed, after adding a non-toxic TLR4 ligand, LpxL1, to the aP vaccine in mice, the *Bordetella pertussis*-specific Th2 response is decreased and Th1/Th17 responses are increased as measured at the cytokine protein level. However, how this shift in Th response by LpxL1 addition is regulated at the gene expression level remains unclear. Transcriptomics analysis was performed on purified CD4^+^ T cells of control and vaccinated mice after *in vitro* restimulation with aP vaccine antigens. Multiple key factors in Th differentiation, including transcription factors, cytokines, and receptors, were identified within the differentially expressed genes. Upregulation of Th2- and downregulation of follicular helper T cell-associated genes were found in the CD4^+^ T cells of both aP- and aP+LpxL1-vaccinated mice. Genes exclusively upregulated in CD4^+^ T cells of aP+LpxL1-vaccinated mice included Th1 and Th17 signature cytokine genes *Ifng* and *Il17a* respectively. Overall, our study indicates that after addition of LpxL1 to the aP vaccine the Th2 component is not downregulated at the gene expression level. Rather an increase in expression of Th1- and Th17-associated genes caused the shift in Th subset outcome.

Pertussis or whooping cough, caused by the gram-negative bacterium *Bordetella pertussis*, remains endemic even in highly vaccinated populations^1^,^2^,^3^. This resurgence has been ascribed to multiple causes, including suboptimal programming of the adaptive immune response by second generation acellular pertussis (aP) vaccines. This has been supported by several studies in different models, namely mice, baboons, and humans, which have revealed that a mixed T helper 1 (Th1) and Th17 type of CD4^+^ T cell response is induced by *B. pertussis* infection^4^,^5^,^6^,^7^. Moreover, these Th subsets have been shown by both the mice and baboon models to be crucial in the protection against *B. pertussis*^4^,^7^. In contrast, the CD4^+^ T cell response induced by current aP vaccines is rather Th2-dominated^4^,^8^,^9^,^10^.

Th subsets are mainly identified by the production of Th subset signature cytokines, such as IFNγ (Th1), IL-4, IL-5, and IL-13 (Th2), IL-17A (Th17), IL-10 and TGFβ (regulatory T cells (Treg)), and IL-21 (follicular helper T cells (Tfh)). CD4^+^ T cell differentiation has several underlying processes. After activation through their T cell receptors, the functional programming of CD4^+^ T cells is initiated by differentiation cytokines produced in the priming microenvironment, such as IL-12, interacting with their cognate receptors. This results in the activation of signal transducer and activator of transcription (Stat) proteins^11^, which induce the expression of master transcription factors. Each Th subset can be defined by the expression of Stat proteins and master transcription factors, namely Stat4/Stat1/Tbet (Th1), Stat5/Stat6/Gata3 (Th2), Stat3/Rorγt (Th17), Stat5/FoxP3 (Treg), and Stat3/Bcl6 (Tfh)^11^,^12^,^13^. These master transcription factors subsequently induce expression of many Th subset-associated genes and silence genes expressed in other Th subsets. These genes include chemokine and cytokine receptors, which also can be used to discriminate between Th subsets. Th1 cells are characterized by CCR1/CCR5/CXCR3 expression, Th2 cells by CCR3/CCR4/CCR8 expression, Th17 cells by CCR4/CCR6 expression, Treg cells by CD25 expression, and Tfh cells by CXCR5 expression^14^.

Recently, the programming of aP vaccine-induced CD4^+^ memory T cells was investigated using genome-wide gene expression profiling of human CD4^+^ T cells^15^. This approach revealed co-expression of both Th2- and Th1-associated gene modules in reactivated CD4^+^ memory T cells generated after aP vaccination in children. This raised the question of how these in principle antagonistic gene modules can establish a predominantly functional Th2 type of CD4^+^ T cell outcome. These gene modules, it was suggested, may exist in a dynamic equilibrium, and depending on ongoing response, the intensity of module components may tip the balance in Th subset outcome towards a Th1 or Th2 response. As several preclinical studies have demonstrated, steering the aP vaccine-induced Th2-dominated response towards a more favorable Th1 and Th17 type of response at the cytokine protein level through the use of adjuvants is feasible, for example through replacement of the currently used adjuvant alum in the aP vaccine with TLR2 or TLR9 ligands^4^,^16^. We recently showed that also adding the TLR4 ligand LpxL1, a non-toxic *Neisseria meningitidis* LPS derivative, to an alum-containing aP vaccine skewed the vaccine-induced CD4^+^ T cell response towards a Th1/Th17 type of CD4^+^ T cell response at the cytokine level^10^. Yet, how the Th subset outcome in the aP vaccine-induced *B. pertussis*-specific CD4^+^ T cell response by LpxL1 as adjuvant is regulated at the level of gene expression remains unclear. This insight is necessary to understand shortcomings and improvement of current aP vaccination.

Therefore, in the present study we compared, in mice, gene expression profiles of *B. pertussis*-specific CD4^+^ T cells induced by aP or LpxL1-adjuvanted aP vaccination. Short stimulation of splenocytes of vaccinated mice with *B. pertussis* antigens activated the *B. pertussis*-specific CD4^+^ T cells, after which microarray analysis was performed on RNA from isolated CD4^+^ T cells. Distinct profiles in CD4^+^ T cells were found that are potentially useful in the evaluation of new vaccine candidates and adjuvants.

## Results

### *B. pertussis*-specific CD4^+^ T cell transcriptome of aP- or aP+LpxL1-vaccinated versus control mice

**Figure 1:**
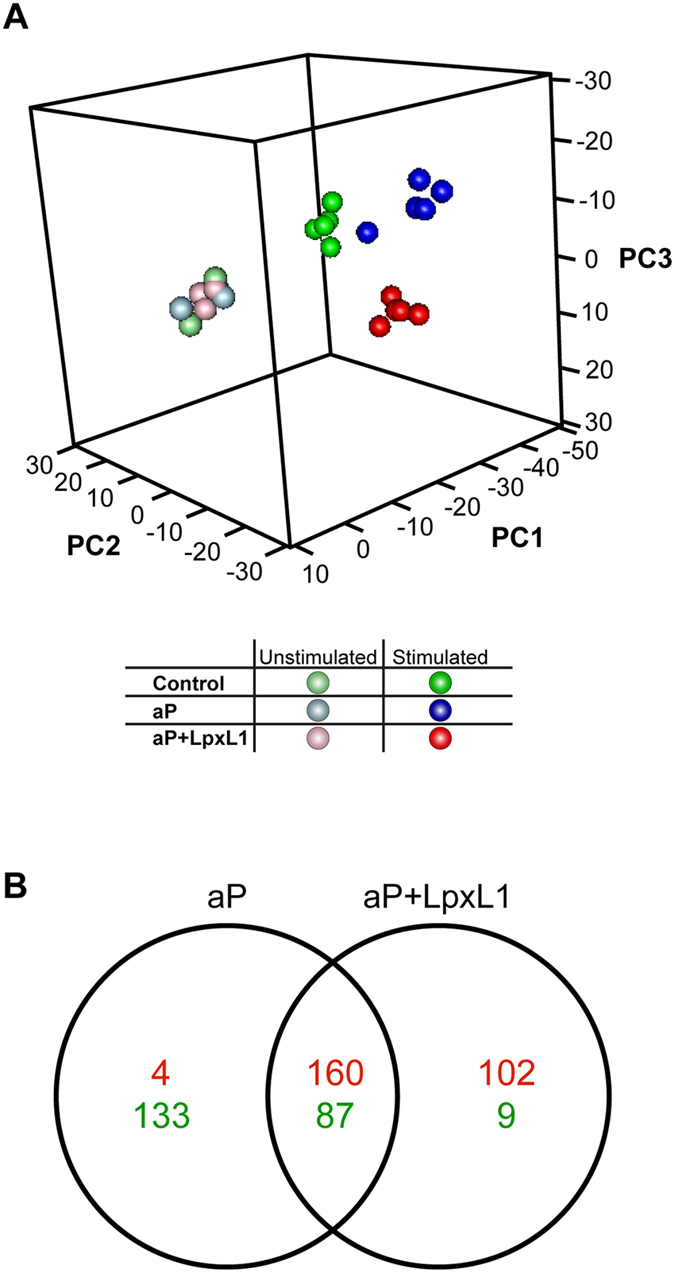
Visualization of differences in gene expression in CD4^+^ T cells of control, aP-, and aP+LpxL1-vaccinated mice by principle component analysis. (**A**) Principal component analysis, based on the differentially expressed genes, showing (dis)similarities in gene expression in samples stimulated with the Ptx, FHA, and Prn combination (dark colors, n = 5 per group) and medium controls (light colors, n = 3 per group) in all vaccination groups (PBS (blue), aP (red), aP+LpxL1 (green)) are shown. (**B**) Venn diagram showing the amount of overlap between up- (red) and downregulated (green) genes in 24 hour *B. pertussis* antigen-stimulated CD4^+^ T cells of aP- and aP+LpxL1-vaccinated mice, as compared to control mice, based on averaged normalized gene expression levels of groups.

**Figure 2:**
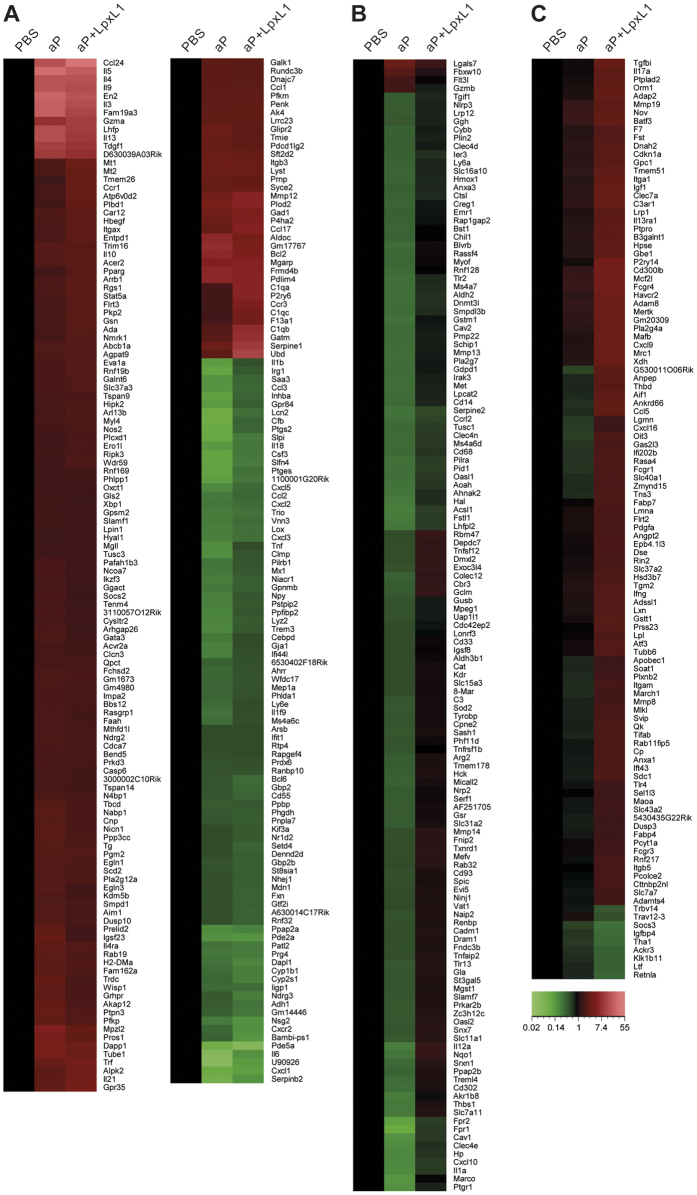
Gene expression profiles of *B. pertussis*-specific CD4^+^ T cells of aP- and aP+LpxL1-vaccinated mice. The heatmaps depict differential up- (red) or downregulation (green) of genes observed in 24 hour *B. pertussis* antigen-stimulated CD4^+^ T cells of vaccinated compared to control mice (FR ≥ 1.5). (**A**) 247 genes were differentially expressed in CD4^+^ T cells of both aP- and aP+LpxL1-vaccinated mice. (**B**) 137 genes were differentially expressed in CD4^+^ T cells of exclusively aP-vaccinated mice. (**C**) 111 genes were differentially expressed in CD4^+^ T cells of exclusively aP+LpxL1-vaccinated mice. Expression data shown are averages from the samples of 5 mice per group.

To determine how addition of LpxL1 to the aP vaccine regulates the Th subset outcome of the vaccine-induced *B. pertussis*-specific CD4^+^ T cells on the molecular level, gene expression profiles of these responding CD4^+^ T cells were investigated. Splenocytes from control, aP-, and aP+LpxL1-vaccinated mice were shortly stimulated with *B. pertussis* antigen Ptx, FHA, and Prn, after which microarray analysis was performed on RNA from isolated CD4^+^ T cells. The gene expression profiles of unstimulated CD4^+^ T cells of all groups were taken as a baseline, to establish whether there is an intrinsic difference between the groups. No significant differentially expressed genes could be identified between these unstimulated samples (criteria: p-value ≤ 0.001, fold ratio (FR) ≥1.5). Nevertheless, to exclude small intrinsic non-significant differences, the expression intensities of the antigen-stimulated samples were corrected for the average expression intensities of unstimulated samples of their corresponding group. In total, 1876 differentially expressed genes (*p*-value ≤ 0.001, FR ≥ 1.5) were identified between averaged unstimulated samples and antigen-stimulated samples of the control, aP-, or aP+LpxL1-vaccinated groups. A principal component analysis on these genes showed differences in gene expression profiles between unstimulated and stimulated samples of all groups, including control mice, suggesting an effect of the stimulation on naive CD4^+^ T cells (Fig. 1A). However, distinct gene expression profiles between stimulated samples of all groups were still observed, revealing functionally differently programmed *B. pertussis-*specific CD4^+^ T cells (Fig. 1B). After comparing the *B. pertussis* antigen-stimulated samples of vaccinated mice with those of control mice, differential expression (FR ≥ 1.5) of 384 and 358 genes was identified in the CD4^+^ T cells of respectively aP- and aP+LpxL1-vaccinated mice. Overlap comparison showed that 247 genes were differentially expressed in CD4^+^ T cells of both aP- and aP+LpxL1-vaccinated mice, 137 genes were exclusively differentially expressed in CD4^+^ T cells of aP-vaccinated mice, and 111 genes were exclusively differentially expressed in CD4^+^ T cells of aP+LpxL1-vaccinated mice (Figs 1 and 2).

### Over-representation of immune and metabolism related terms after aP- and aP+LpxL1- vaccination

**Figure 3:**
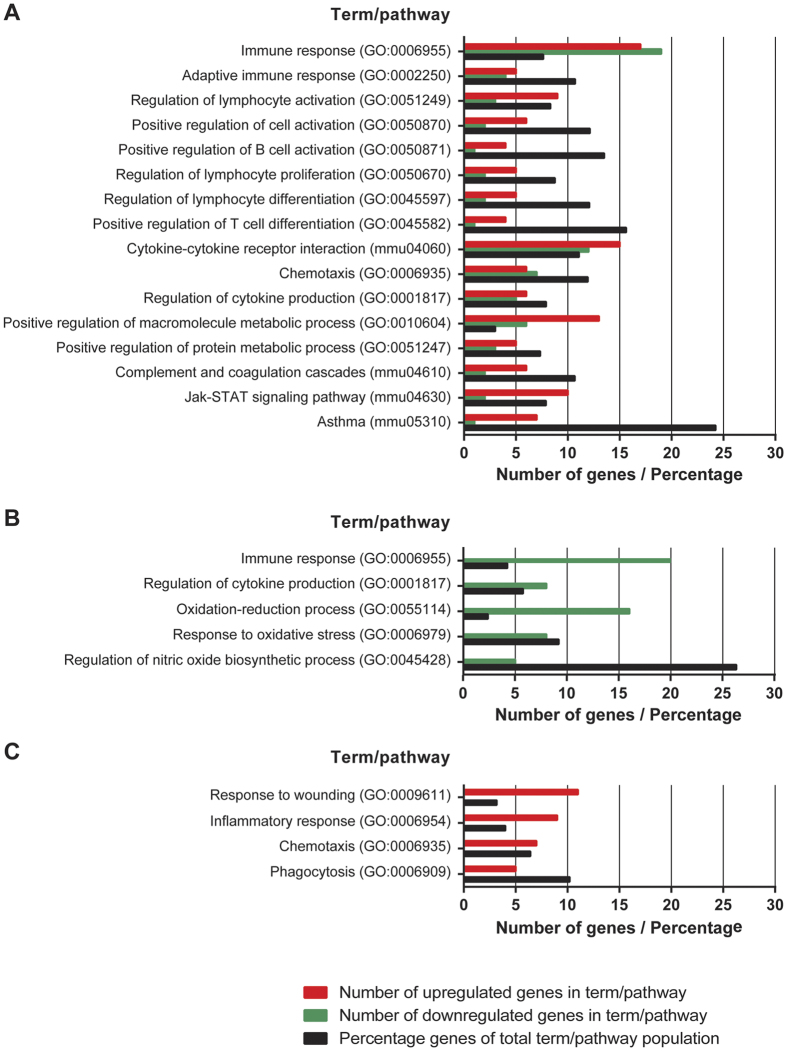
Gene expression profiles of *B. pertussis*-specific CD4^+^ T cells of aP- and aP+LpxL1-vaccinated mice. Functional annotation and pathway enrichment of differentially expressed genes in *B. pertussis*-specific CD4^+^ T cells of aP- and aP+LpxL1-vaccinated mice. Over-representation analysis (Benjamini-corrected *p*-value ≤ 0.05) in GO-BP and KEGG databases was performed using genes differentially expressed in *B. pertussis* antigen-stimulated CD4^+^ T cells of vaccinated compared to control mice. Functional annotation and pathway enrichment are depicted from genes differentially expressed in CD4^+^ T cells of both aP- and aP+LpxL1 vaccinated mice (**A**), in CD4^+^ T cells of exclusively aP-vaccinated mice (**B**), and in CD4^+^ T cells of exclusively aP+LpxL1-vaccinated mice (**C**). The amount of up- or downregulated genes per term/pathway and the percentage of the genes in the total term/pathway population are shown.

To provide more insight in the differentially expressed genes, functional annotation and over-representation analysis (Benjamini-corrected p-value ≤ 0.05) in GO-BP and KEGG databases were performed using DAVID^17^. Analysis of the overlapping 247 differentially expressed genes in CD4^+^ T cells of both aP- and aP+LpxL1-vaccinated mice showed that 74 GO-BP terms and 8 KEGG pathways were enriched. Based on exclusion of overlapping terms/pathways and their relevance, a selection of these terms/pathways is shown in Fig. 3A. The enriched terms/pathways are mainly involved in the regulation of the adaptive immune response, as indicated by terms as regulation of lymphocyte activation (GO:0051249), proliferation (GO:0050670), and differentiation (GO:0045597), and cytokine signaling, including chemotaxis (GO:0006935) and Jak-STAT signaling pathway (mmu4630). Moreover, the enrichment of the asthma pathway (mmu05310) indicates the presence of Th2-associated genes. Further, terms involved in metabolic processes are enriched, including positive regulation of macromolecule metabolic process (GO:0010604) and positive regulation of protein metabolic process (GO:0051247).

Functional annotation and over-representation analysis (Benjamini-corrected *p*-value ≤ 0.05) of the 137 genes differentially expressed in CD4^+^ T cells of exclusively aP-vaccinated mice revealed enrichment of 9 GO-BP terms. Five relevant terms are depicted in Fig. 3B, which includes immune response-related terms, such as immune response (GO:0006955) and regulation of cytokine production (GO:0001817), and metabolism-related terms such as oxidation-reduction process (GO:0055114) and regulation of nitric oxide biosynthetic process (GO:0045428). Functional annotation and over-representation analysis of the 111 genes solely altered in CD4^+^ T cells of aP+LpxL1-vaccinated mice showed enrichment of 9 GO-terms, including inflammatory response (GO:0006954), chemotaxis (GO:0006935), and phagocytosis (GO:0006909) (Fig. 3C).

### Differential expression of cytokine-encoding genes in vaccine-induced CD4^+^ T cells

**Figure 4:**
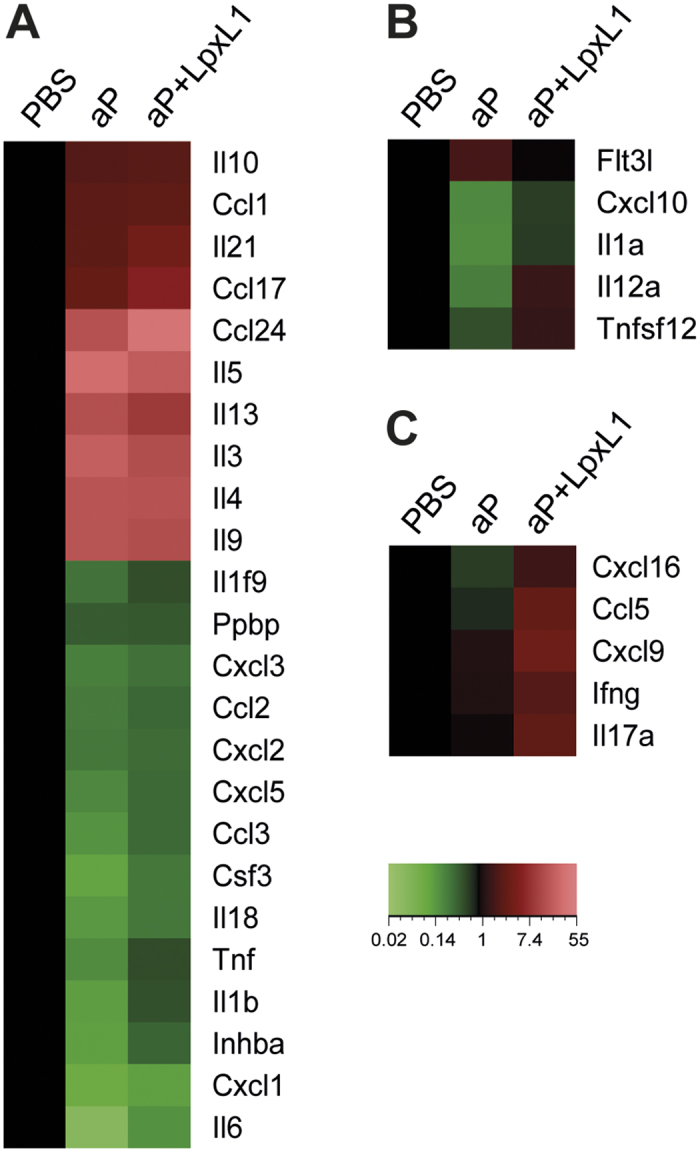
Gene expression profile of cytokine encoding genes in *B. pertussis*-specific CD4^+^ T cells of aP- and aP+ LpxL1-vaccinated mice. Genes encoding cytokines differentially expressed in *B. pertussis* antigen-stimulated CD4^+^ T cells of both aP- and aP+LpxL1-vaccinated mice (**A**), in CD4^+^ T cells of exclusively aP-vaccinated mice only (**B**), and in CD4^+^ T cells of exclusively aP+LpxL1-vaccinated mice only (**C**). Expression data shown are averages from the samples of 5 mice per group.

Our previous study investigated the type of CD4^+^ T cell response at the protein level by determining the percentage of *B. pertussis* antigen-specific IL-5-, IFNγ-, and IL-17A-positive CD4^+^ T cells using flow cytometry and by supernatant analysis. It showed that addition of LpxL1 to the aP vaccine skews the CD4^+^ T cell response of a Th2-dominated to a mixed response, dominated by Th1/Th17^10^. Therefore, we investigated in more detail the expression of cytokine-encoding genes. Some Th subset signature cytokine-encoding genes could be identified which were upregulated in the CD4^+^ T cells of both aP- and aP+LpxL1-vaccinated mice, such as *Il4*, *Il5*, *Il13, Il21*, and *Il10* (Fig. 4A). No signature cytokine-encoding genes were found to be differentially expressed in the CD4^+^ T cells of solely aP-vaccinated mice, while both *Ifng* and *Il17a* were found to be upregulated exclusively in those of aP+LpxL1-vaccinated mice (Fig. 4C). In addition to the Th subset signature cytokines-encoding genes, other cytokine genes were differentially expressed of which 19 were found in CD4^+^ T cells of aP- as well as aP+LpxL1-vaccinated mice. Genes *Il3*, *Il9*, *Ccl1*, *Ccl17*, and *Ccl24* were upregulated, whereas downregulation was found for genes encoded for chemokines, *Cxcl1*, *Ccl2*, *Cxcl2*, *Cxcl5*, *Cxcl3*, *Ccl3*, and *Csf3*, and pro-inflammatory cytokines, *Il1b*, *Il6*, *Tnf*, and *Il18* (Fig. 4A). Five genes encoding other cytokines were detected in the CD4^+^ T cells of exclusively aP-vaccinated mice, which included downregulation of *Cxcl10*, *IL12a*, *Il1a*, and *Tnfsf12* and upregulation of *Flt3l* (Fig. 4B). Three upregulated genes were found only in those of aP+LpxL1-vaccinated mice, namely *Cxcl9*, *Ccl5*, and *Cxcl16* (Fig. 4C). Together, these results indicate substantial overlap in the expression of cytokine-encoding genes, including Th2 signature cytokines, after both aP- and aP+LpxL1- vaccination, while expression of genes encoding Th1 and Th17 signature cytokines is only induced by aP+LpxL1 vaccination.

### Differential expression of transcription factor-encoding genes in vaccine-induced CD4^+^ T cells

**Figure 5:**
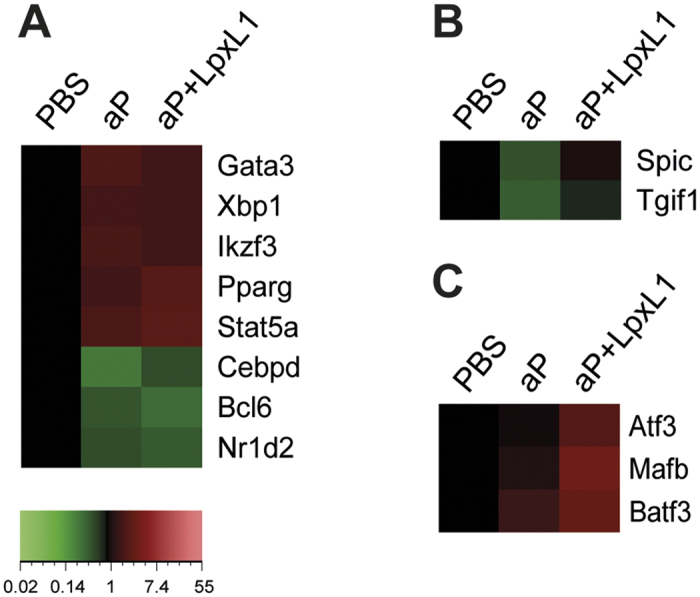
Gene expression profile of transcription factor encoding genes in *B. pertussis*-specific CD4^+^ T cells of aP- and aP+LpxL1-vaccinated mice. Genes encoding transcription factors differentially expressed in *B. pertussis* antigen-stimulated CD4^+^ T cells of both aP- and aP+LpxL1-vaccinated mice (**A**), in CD4^+^ T cells of exclusively aP-vaccinated mice only (**B**), and in CD4^+^ T cells of exclusively aP+LpxL1-vaccinated mice only (**C**). Expression data shown are averages from the samples of 5 mice per group.

Important in the differentiation of CD4^+^ T cells to different Th subsets are the master transcription factors, T-bet, Gata3, Rorγt, Bcl6, and FoxP3^11^,^12^,^13^. Within the CD4^+^ T cells of both aP- and aP+LpxL1-vaccinated mice, *Gata3*, the gene encoding the Th2 master transcription factor was found upregulated whereas *Bcl6,* the gene encoding the Tfh master transcription factor was found downregulated (Fig. 5A). Genes encoding other known master transcription factors were not found differentially expressed. The expression of master transcription factors is regulated by different Stat proteins^11^. Upregulation of only one Stat gene, namely *Stat5a*, which is involved in the differentiation of Th2 and Treg cells, was detected within CD4^+^ T cells of both aP- and aP+LpxL1-vaccinated mice (Fig. 5A). In addition, five genes encoding other transcription factors were identified as differentially expressed in the CD4^+^ T cells of both aP- and aP+LpxL1-vaccinated mice, including upregulation of *Pparg*, *Xbp1*, and *Ikzf3* and downregulation of *Nrld2* and *Cebpd* (Fig. 5A). Transcription factors *Spic* and *Tgif1* were found downregulated only in the CD4^+^ T cells of the aP-vaccinated mice (Fig. 5B), while transcription factors *Atf3*, *Mafb* and *Batf3* were found upregulated only in the CD4^+^ T cells of aP+LpxL1-vaccinated mice (Fig. 5C). Based on expression of Th differentiating transcription factors, both aP- and aP+LpxL1 vaccination induce Th2 and inhibit Tfh differentiation.

### Differential expression of receptor- and cell surface molecule-encoding genes in vaccine-induced CD4^+^ T cells

**Figure 6:**
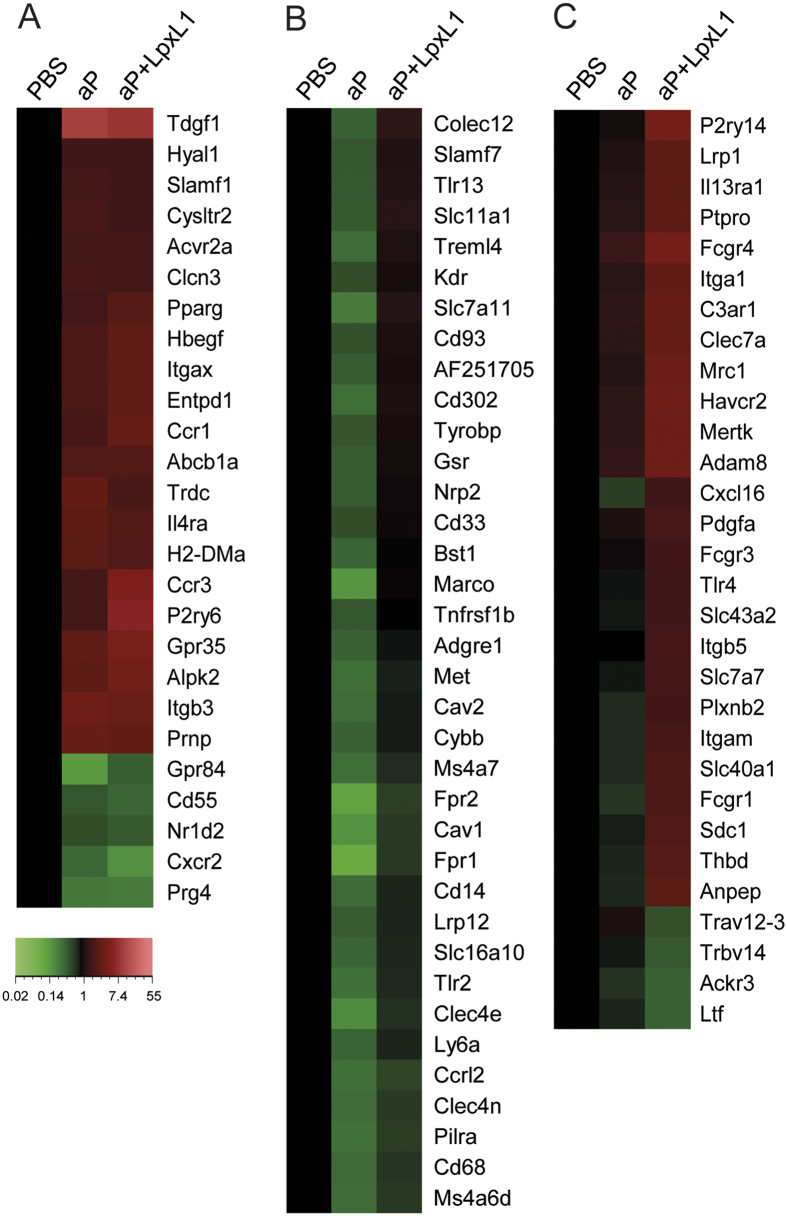
Gene expression profile of genes encoding receptors and cell surface markers in *B. pertussis*-specific CD4^+^ T cells of aP- and aP+LpxL1-vaccinated mice. Genes encoding receptors and cell surface markers differentially expressed in *B. pertussis* antigen-stimulated CD4^+^ T cells of both aP- and aP+LpxL1-vaccinated mice (**A**), in CD4^+^ T cells of exclusively aP-vaccinated mice only (**B**), and in CD4^+^ T cells of exclusively aP+LpxL1-vaccinated mice only (**C**). Expression data shown are averages from the samples of 5 mice per group.

Another way to characterize CD4^+^ T cell subsets is by the expression of certain receptors and cell surface markers. Upregulation of markers *Ccr1* and *Ccr3* was detected in the CD4^+^ T cells of aP- and aP+LpxL1-vaccinated mice (Fig. 6A). Remarkably, higher expression of the Th2-associated *Ccr3* was seen in aP+LpxL1 samples than in aP samples (Fig. 6A). In addition to the markers used to characterize Th subsets, differential expression was found of genes encoding other receptors and cell surface molecules. Of these genes, 24 were found in CD4^+^ T cells of aP- and aP+LpxL1-vaccinated mice, and of these, 19 genes were upregulated, including *Il4ra,* and 5 genes were downregulated, including *Cxcr2* (Fig. 6A). Within the CD4^+^ T cells of aP-vaccinated mice, 36 receptor- and cell surface marker-encoding genes were downregulated, including *Ly6a*, and multiple genes encoding for proteins involved in pattern recognition, like *Tlr2*, *Tlr13*, *Clec4a*, *Clec4n*, and *Cd14* (Fig. 6B). The 26 upregulated receptor- and cell surface marker-encoding genes in the CD4^+^ T cells of aP+LpxL1-vaccinated mice included *Havcr2, Itga1,* and genes encoding proteins involved in the innate immune response, such as *Tlr4*, *Clec7a*, *C3ar1*, *Fcgr1*, *Fcgr3*, and *Fcgr4* (Fig. 6C). The 4 downregulated receptor- and cell surface marker-encoding genes in samples of aP+LpxL1-vaccinated mice were *Ackr3, Ltf, Trbv14*, and *Trav12-3*. Together, these results suggest that aP+LpxL1 vaccination induces expression of genes encoding receptors and cell surface markers associated with Th2 (*Ccr3* and *Il4ra*), Th1 (*Havcr2*), and Th17 (*Il13ra1*) subsets, while aP vaccination only induced genes associated with the Th2 (*Ccr3* and *Il4ra*) subset.

### Differential expression of genes encoding proteins involved in metabolism in vaccine-induced CD4^+^ T cells

**Figure 7:**
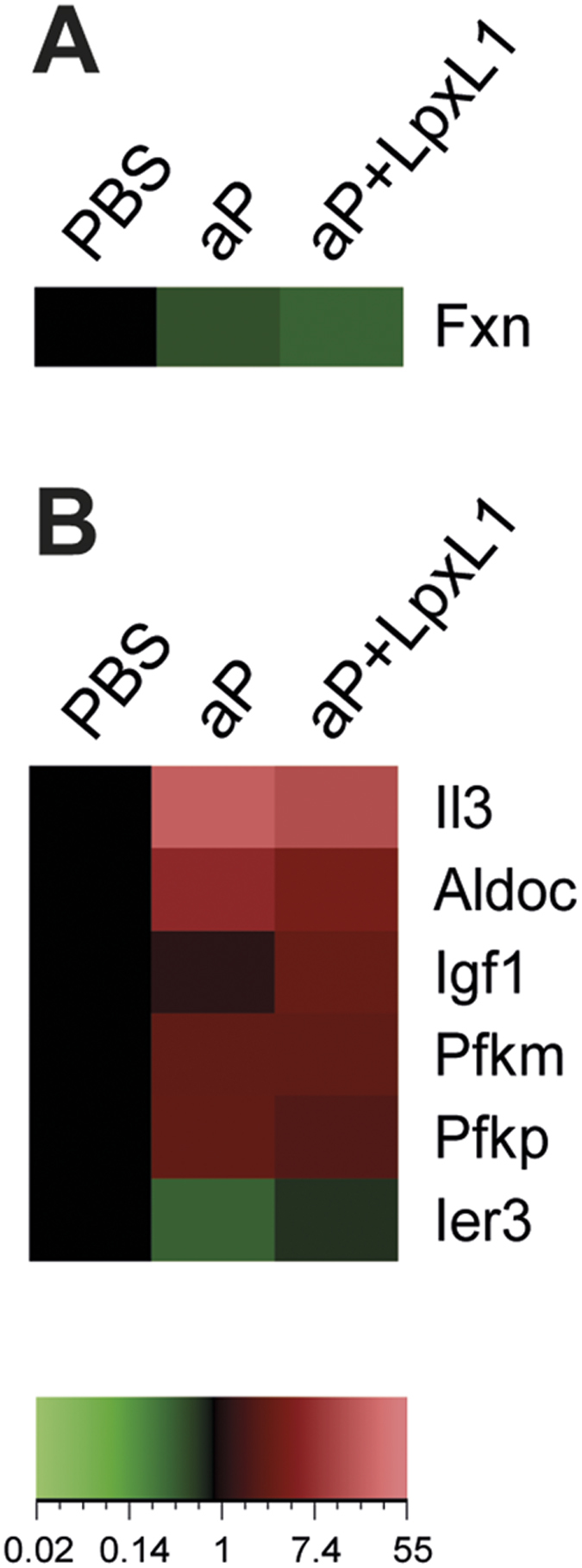
Gene expression profile of genes encoding proteins involved in metabolism in *B. pertussis*-specific CD4^+^ T cells of aP- and aP+LpxL1-vaccinated mice. Heatmaps depict genes involved in oxidative phosphorylation (**A**) and glycolytic process (**B**) that are differentially expressed in *B. pertussis* antigen-stimulated CD4^+^ T cells of aP- and aP+LpxL1-vaccinated mice compared to control mice. Expression data shown are averages from the samples of 5 mice per group.

Recent studies have revealed that a shift in metabolism from oxidative phosphorylation toward aerobic glycolysis is important in the activation of T cells^18^. Moreover, the production of IFNγ in effector T cells requires aerobic glycolysis^19^. For this reason we also analyzed the expression of genes involved in these metabolic pathways. Only one gene encoding a protein involved in the oxidative phosphorylation was found differentially expressed, namely *Fxn*. The *Fxn* gene was downregulated in CD4^+^ T cells of both aP- and aP+LpxL1-vaccinated mice (Fig. 7A). Additionally, six genes encoding for proteins with a function in the glycolytic process could be identified in the CD4^+^ T cells (Fig. 7B). Four genes were found upregulated in both vaccinated groups, namely *Aldoc*, *Il3*, *Pfkm*, and *Pfkp*. The *Ier3* gene was downregulated in CD4^+^ T cells of aP-vaccinated mice, while *Igf1* was upregulated in those of aP+LpxL1-vaccinated mice. In addition, a recent study has shown that regulation of glucose uptake induced by Notch signaling is important in the survival of memory CD4^+^ T cells^20^. However, no genes involved in this pathway were found to be differentially expressed in the CD4^+^ T cells of aP- and aP+LpxL1-vaccinated mice. Overall, these data suggest that there is no difference in the expression of genes involved in metabolic pathways in CD4^+^ T cells of aP- and aP+LpxL1-vaccinated mice.

### Distinct Th subset-associated gene modules expressed after aP- and aP+LpxL1 vaccination

**Figure 8:**
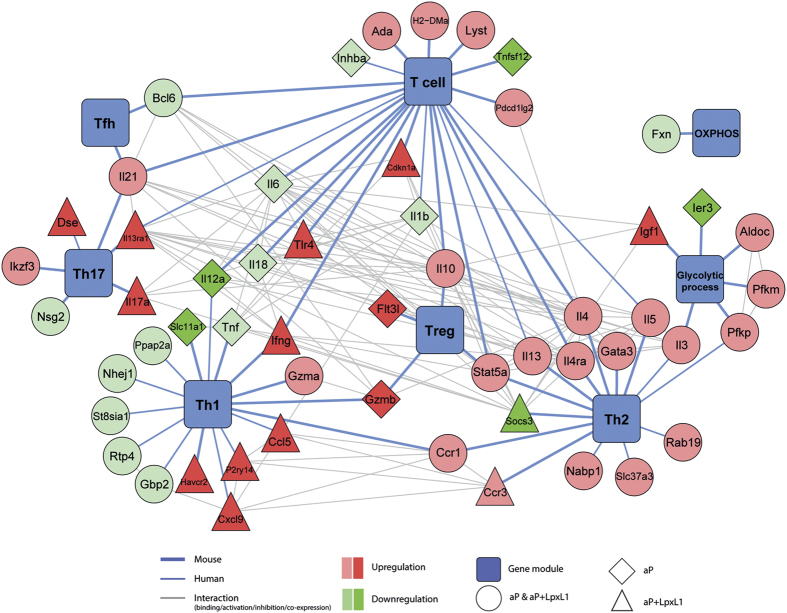
Network analysis of Th subset-associated genes differentially expressed in *B. pertussis*-specific CD4^+^ T cells of aP- and aP+LpxL1-vaccinated mice. A gene-function network analysis showing the Th subset-associated genes differentially expressed in *B. pertussis* antigen-stimulated CD4^+^ T cells of aP- and aP+LpxL1-vaccinated was performed using Cytoscape to visualize the patterns of Th subset-associated genes induced by the different vaccines. Association of genes with the gene modules (blue rectangles) was based on literature from mouse (bold blue lines) and human (thin blue lines) studies. The interactions between genes (grey lines) were determined using the STRING database. The shape of the gene nodes indicate whether genes were differentially expressed in CD4^+^ T cells of both vaccination groups (circles), had the highest fold-change in either the CD4^+^ T cells of aP-vaccinated mice (diamonds) or in those of aP−LpxL1-vaccinated mice (triangles). The color intensity of the gene nodes indicate whether genes were differentially expressed in CD4^+^ T cell of both aP- and aP+LpxL1-vaccinated mice (light green and red) or in CD4^+^ T cells of exclusively aP-vaccinated mice or in those of exclusively aP+LpxL1-vaccinated mice (dark green and red).

Based on literature from human and murine studies, a network analysis was performed to visualize the expression patterns of genes associated with different Th subsets that were observed in the CD4^+^ T cells of aP- and aP+LpxL1-vaccinated mice (Fig. 8). In addition to genes encoding the previously mentioned master transcription factors, signature cytokines, and surface markers, other differentially expressed genes associated with the main Th subsets were found. Mainly Th2-associated genes, such as the Th2 subset signature cytokines (*Il4*, *Il5*, and *Il13*), *Gata3*, *Il3*, *Nabp1*, and *Slc37a3*, were found upregulated in the CD4^+^ T cells of both aP- and aP+LpxL1-vaccinated mice. Interestingly, another Th2-associated gene, *Socs3*, was downregulated in the CD4^+^ T cells of exclusively aP+LpxL1-vaccinated mice. Th1-associated genes were upregulated in CD4^+^ T cells of aP+LpxL1-vaccinated mice, including *Havcr2* and chemokines *Cxcl9* and *Ccl5*, while downregulation of Th1-associated genes *Scl11a1* and *Il12a* is observed in those of aP-vaccinated mice. Further, upregulation of Th17-associated genes *Dse*, *Il13ra1*, and *Il17a* was only observed in the CD4^+^ T cells of aP+LpxL1-vaccinated mice. Differential expression of Treg-associated genes was found in the CD4^+^ T cells of aP- as well as aP+LpxL1-vaccinated mice, namely *Il10* and *Stat5a*. However, other Treg-associated genes, *Flt3l* and *Gzmb*, were only upregulated in CD4^+^ T cells of aP-vaccinated mice. Only 2 Tfh-associated genes were found in our study, *Bcl6* and *Il21*, which were respectively down- and upregulated in CD4^+^ T cells of both the aP- and aP+LpxL1-vaccinated mice. Moreover, genes involved in glycolysis were found in CD4^+^ T cells of both vaccination groups of which 2 genes are associated with the Th2 subset, namely *Pgkp* and *Il3*. Based on this gene expression network, our results suggest that aP vaccination induces mainly Th2 and Treg gene modules, while addition of LpxL1 to the aP vaccine induces a shift towards Th1 and Th17 gene modules.

### Enrichment of transcription factor-binding sites within the gene set of differentially expressed genes in CD4^+^ T cells of aP- or aP+LpxL1-vaccinated mice

To further provide insight in the concerted regulation of the differentially expressed genes in CD4^+^ T cells of aP- and aP+LpxL1 mice, a transcription factor-binding site (TFBS) analysis was performed. This analysis revealed enrichment of binding sites for SPIB, RELA, and IRF2 within the promoter regions of upregulated genes in the CD4^+^ T cells of aP-vaccinated mice and ELF5, SPI1, Klf4, SPIB, RELA, REL, ELK1, NF-kappaB, and FEV within the upregulated genes in the CD4^+^ T cells of aP+LpxL1-vaccinated mice, respectively (Supplementary Figure S1). Binding sites for transcription factors within the downregulated genes in the CD4^+^ T cells of aP-vaccinated mice were NF-kappaB and RELA, while no enrichment of TFBS was found within the downregulated genes in the CD4^+^ T cells of aP+LpxL1-vaccinated mice (Supplementary Figure S1). An overview of the top 20 transcription factors from each analyzed gene set is given in Supplementary Table S1. These results suggest the involvement of multiple transcription factors that regulate the distinct Th subset-related gene expression observed after addition of LpxL1 to the aP vaccine. Whereas SPIB and RELA were found in both groups, SPI1, Klf4, and NF-kappaB were only involved after addition of Lpxl1.

## Discussion

Addition of the TLR4 ligand LpxL1 to an aP vaccine was found to dampen the Th2 dominance of the antigen-specific CD4^+^ T cell response of vaccinated mice and to increase a Th1/Th17 type response, based on cytokine analysis^10^. In the present study, this skewing was investigated in more detail at the gene expression level. Analysis of the expression of Th subset signature cytokine-encoding genes revealed an increased expression of *Ifng* and *Il17a* in CD4^+^ T cells of exclusively aP+LpxL1-vaccinated mice, which is consistent with our previous findings. Most importantly, the Th2 subset signature cytokine genes *Il4*, *Il5*, and *Il13* showed increased expression in the CD4^+^ T cells of aP- as well as aP+LpxL1-vaccinated mice, suggesting that the Th2 component is not downregulated at the gene expression level of Th subset signature cytokines after addition of LpxL1 to the aP vaccine.

Other Th1-, Th17-, and Th2-associated genes showed the same trend as the genes encoding Th signature cytokines. Genes associated with the Th1 subsets had increased expression in the CD4^+^ T cells of exclusively aP+LpxL1-vaccinated mice, including genes encoding chemokines (*Ccl5* and *Cxcl9*) and cell surface marker *Havcr2* (*Tim3*). Both Ccl5 and Cxcl9 are chemoattractants for Th1 cells and are described to be produced by human CD4^+^ T cells^21^,^22^. Havcr2 is a cell surface marker preferentially expressed on Th1 cells and its expression is induced by Th1 master transcription factor T-bet^23^. Th17-associated genes that showed increased expression solely in the CD4^+^ T cells of aP+LpxL1-vaccinated mice were *Il13ra1* and *Dse*. IL-13Rα1 is a functional receptor found on both murine and human Th17 cells while it is not expressed on Th0, Th1, Th2, and Treg cells^24^. Binding of IL-13 to this receptor attenuates the production of IL-17A^24^. Further, Dse is an intracellular enzyme involved in epitope processing and is preferentially expressed in human Th17 cells^25^.

Remarkably, several other Th2-associated genes also showed increased expression in CD4^+^ T cells of both aP- and aP+LpxL1-vaccinated mice, of which most genes showed the same trend as the expression of the Th2 subset signature cytokine-encoding genes. These other Th2-associated genes include Th2 master transcription factor *Gata3*, Stat protein *Stat5a*, chemokine-receptors *Ccr1* and *Ccr3*, and cytokine-receptor *Il4ra*, and other genes, namely *Rab19*, *Nabp1*, *Scl37a3*, and *Pfkp*^15^. Interestingly, downregulation of Th2-associated *Socs3* is observed in the CD4^+^ T cells of exclusively aP+LpxL1-vaccinated mice. Socs3, suppressor of cytokine signaling-3, is preferentially expressed in Th2 cells^26^ and inhibits Th1 and Th17 differentiation by suppressing STAT4 and STAT3 activation, respectively^27^,^28^. Downregulation of *Socs3* in CD4^+^ T cells of aP+LpxL1-vaccinated mice suggests reduced active suppression of Th1 and Th17 differentiation when LpxL1 is present in the aP vaccine and thereby favors Th1 and Th17 differentiation.

In addition to the involvement of the Th1, Th2, and Th17 subsets, this study in aP- and aP+LpxL1-vaccinated mice revealed gene expression modules pointing at the induction or inhibition of other Th subsets, namely Treg and Tfh. Treg cells were induced by both aP- and aP+LpxL1 vaccination, since increased expression of a Treg subset signature cytokine gene, *Il10*, as well as the Treg-associated Stat gene, *Stat5a*, was detected in CD4^+^ T cells of both groups. However, expression of *Gzmb*, encoding Granzyme B, which has cytolytic functions and is expressed in different cells including Tregs^29^,^30^, showed increased expression in the CD4^+^ T cells of exclusively aP-vaccinated mice. Together with the increased expression of *Flt3l*, which is involved in the expansion of Treg cells^31^, in only the samples of aP-vaccinated mice, this suggests that increased numbers of Treg cells were induced after vaccination with the aP- vaccine alone. Tfh master transcription factor *Bcl6* showed decreased expression in the CD4^+^ T cells of both aP- and aP+LpxL1-vaccinated mice, indicating that differentiation towards the Tfh subset was suppressed. This seems contradictory given the increased expression of the Tfh subset signature cytokine gene *Il21*. However, this cytokine can also be produced by Th17 cells^32^. Some induction of Th17 cells by aP vaccination might explain the increased expression of *Il21*. A study of Ross *et al.* indeed showed that Th17 cells could be detected in mice after aP vaccination^4^. These results are consistent with the increased expression of *Ikzf3* in CD4^+^ T cells of both aP- and aP+LpxL1-vaccinated mice, since this gene is specifically expressed in Th17 cells^33^.

In addition to Th subset-associated genes, genes encoding proteins that are involved in metabolism were investigated, since a shift in metabolism from oxidative phosphorylation toward aerobic glycolysis is important in the activation of T cells^18^. Only a small number of genes involved in oxidative phosphorylation and glycolysis were differentially expressed in the CD4^+^ T cells of aP- and aP+LpxL1-vaccinated mice. The genes encoding proteins involved in glycolysis, namely *Il3, Pfkp*, *Aldoc,* and *Pfkm* showed increased expression in the samples of both aP- and aP+LpxL1-vaccinated mice. Interestingly, *Il3* and *Pfkp* are also associated with Th2 cells^15^. Overall, these results suggest little or no difference in the activation of CD4^+^ T cells based on metabolism by the different vaccines.

Within the set of genes differentially expressed in CD4^+^ T cells of aP- and aP+LpxL1-vaccinated mice, genes were found encoding proteins with a known function in the innate immune system, including cytokines (*Il6, Il1b, Tnf*, and *Il12a*), complement components (*C1qa*, *C1qb*, *C1qc*, *Cd55*, *Cfb, C3*, *Cd93*, *C3ar1, Itgam*), Toll-like receptors *(Tlr2*, *Tlr13*, and *Tlr4)*, C-type lectin receptors (*Clec4a*, *Clec4d*, *Clec4n*, *Clec7a*, and *Cd302*), and Fc-receptors (*Fcgr1*, *Fcgr3*, and *Fcgr4*). It is unlikely that these innate gene signatures can be fully explained by contamination of innate immune cells within the CD4^+^ T cell fraction, since the purity of the samples was >95%. Interestingly, several of these innate immunity genes are known to be expressed in CD4^+^ T cells, including complement components such as *Itgam, C3ar1*, and *Cd55*^34^,^35^,^36^. Signaling through C3a receptor 1, upregulated gene in aP+LpxL1 samples, by binding a derivative of C3, downregulated gene in aP samples, has been associated with a Th2^37^ and a Th1 response^38^, and with inhibition of Treg function^39^. Moreover, some TLRs, such as TLR2 and TLR4, are also expressed on CD4^+^ T cells. Signaling via TLR2, which is downregulated in aP samples, has been found to induce IFNγ production by Th1 cells^40^ and might even inhibit IL-4 production^41^. In addition, TLR2 signaling promotes the differentiation of Tregs into Th17 cells in human^42^. Signaling through TLR4 which gene expression is upregulated in aP+LpxL1 samples is reported to provide a signal for proliferation and cell survival and seems to regulate persistence of Th lineages^43^. Furthermore, the Fc-gamma receptor *Fcgr3* gene, which was upregulated in aP+LpxL1 samples, was shown to be expressed on a small proportion of CD4^+^ T cells with an effector memory phenotype^44^ and activated CD4^+^ T cells expressing IFNγ and T-bet^45^. Together these data indicate that the differential expression of innate genes could have a function in CD4^+^ T cells.

TFBS analysis indicated enrichment of binding sites for three members of the NF-κB family, REL, RELA, and NF-kappaB, in the gene set from the CD4^+^ T cells of aP+LpxL1-vaccinated mice, while enrichment of binding sites of only one member, RELA, was observed in those of aP-vaccinated mice. Signaling via multiple receptors, including T cell receptor, TLRs, including TLR4, and pro-inflammatory cytokine receptors, can lead to the activation of NF-κB^46^. Together with the observed upregulation of *Tlr4* in the gene set of CD4^+^ T cells of exclusively aP+LpxL1-vaccinated mice, this suggests that LpxL1 might directly activate these transcription factors via TLR4 signaling. Moreover, there is evidence that and RELA is associated with Th17 differentiation^47^ and REL with Th1^48^ and Th17 differentiation^47^, although conflicting results are published regarding the association of REL with Th17 differentiation^49^. In addition, enrichment of binding sites of Klf4 was observed in the gene set of CD4^+^ T cells of exclusively aP+LpxL1-vaccinated mice, which is also associated with Th17 differentiation^50^. Binding sites for SPI1 were also enriched within this gene set, which is known to inhibit the expression of Th2 cytokines^51^. Together, the data indicate that LpxL1 activates several transcription factors associated with Th1 and Th17 differentiation, which corroborates our findings of the expression of Th-related genes. Furthermore, the results suggest that LpxL1 might activate these transcription factors via TLR4 signaling.

Within the CD4^+^ T cells of aP+LpxL1-vaccinated mice, increased expression of Th1- and Th17-associated genes, including the signature cytokine genes *Ifng* and *Il17a,* was observed. However, no increased expression of the master transcription factors of Th1 and Th17 cells, *Tbx21* and *Rorc* respectively, was found. An *in vitro* effect of 24-hour stimulation with *B. pertussis* antigens might underlie this effect, since in our previous study IFNγ and IL-17A production by naive CD4^+^ T cells was detected after stimulation with the *B. pertussis* antigens^10^. Indeed, in the current study, differently expressed genes found between unstimulated versus antigen-stimulated CD4^+^ T cells of control mice were detected, including the Th1 master transcription factor gene *Tbx21* and Th1 Stat gene *Stat1*. Therefore, we interpret the lack of differential expression of Th1 and Th17 master regulators in the samples of the vaccinated mice compared to those of control mice to be a result of an increased background expression in naive CD4^+^ T cells induced by the *in vitro B. pertussis* antigen stimulation. This *in vitro* activation of naive CD4^+^ T cells could also explain why only a few genes were found corresponding to proteins involved in metabolism, since the metabolism is altered by activation of CD4^+^ T cells^18^.

Although addition of LpxL1 to the aP vaccine led to a decreased percentage of Th2 cells and reduced *in vitro* Th2 cytokine levels in *B. pertussis* antigen-stimulated CD4^+^ T cell cultures of vaccinated mice in our previous study^10^, no or only a limited decrease in expression of Th2-associated genes was observed in the current study, except for *Socs3*. This might be explained by the duration of *in vitro* stimulation of the CD4^+^ T cells, since in the gene expression analysis the duration was shorter (24 hours) than in the functional read-out study (8 days). In addition, there might be reduced translation of the Th2 cytokine mRNA due to Th1- and Th17-associated miRNA translational repression. Such mechanism was shown for Th1-specific miR-135b^52^ repressing Th2-associated genes *Stat6* and *Gata3* mRNA translation to protein^53^. Therefore, we propose that the shift towards a mixed Th1 and Th17 response is likely due to increased expression of Th1- and Th17-associated gene modules rather than downregulation of the Th2-associated gene module. Interestingly, White *et al.* also found a decisive role for the Th1 gene network module in the outcome of Th responses. In their study, extreme Th2 dominance in atopic allergy was associated with the complete absence of the Th1 gene network module^15^. A limitation of our study is that the differences on gene expression are measured on the total splenic CD4^+^ T cell population. Therefore, the question remains whether the shifts in gene modules observed at the population level also occur within the same cell. In future research, investigating the gene expression on single cell level can overcome this limitation, as was described by Chattopadhyay *et al.*^54^.

In summary, this study provides a gene expression network model that may explain why aP vaccination induces Th2 and Treg differentiation of CD4^+^ T cells, and why addition of LpxL1 to the aP vaccine leads to the induction of Th1 and Th17 cells. Together with our previous data, showing a shift from a Th2-dominated response to a mixed Th1/Th17 response at the cytokine protein level, this study indicates that only a small change in the balance between the expression of Th1/Th17- and Th2-associated genes results in a shift in Th type. Moreover, this model can be used in the evaluation of the effects of new adjuvants on vaccination-induced T cell responses, in particular in the context of improving acellular pertussis vaccines.

## Materials and Methods

### Ethics statement

This study was approved by the Committee on Animal Experimentation of the Netherlands Vaccine Institute (Bilthoven, The Netherlands) under permit number 201200115. Animal handling in this study was carried out in accordance with relevant Dutch national legislation, including the 1997 Dutch Act on Animal Experimentation.

### Vaccines and antigens

Pertactin P.69 (Prn) was expressed in *Escherichia coli*, purified as described previously^55^ and was tested for *E. coli* LPS impurities using a Limulus Amebocyte Lysate (LAL) test. The endotoxin level was <0.015 EU/ml. Purified filamentous hemagglutinin (FHA) and pertussis toxin (Ptx) were obtained from Kaketsuken (Japan) and Ptx was heat-inactivated at 95 °C for 15 minutes before use. The registered combined pentavalent diphtheria, tetanus, and acellular pertussis vaccine (Infanrix; aP) was purchased from GlaxoSmithKline and one human dose (HD) contains a minimum of 30 I.E. diphtheria toxoid, a minimum of 40 I.E. tetanus toxoid, 25 μg FHA, 25 μg Ptx, and 8 μg Prn, all absorbed to aluminumhydroxide. LpxL1, a meningococcal LPS derivative, was engineered and obtained as described elsewhere^56^.

### Mice and immunization

Adult (6–8 weeks old) Balb/c mice (Harlan, The Netherlands) were vaccinated s.c. on day 0 (right flank) and day 28 (left flank) with 0.3 ml of 1/4 HD aP vaccine, 1/4 HD aP vaccine supplemented with 1 μg non-adsorbed LpxL1 (aP+LpxL1), or as a control with PBS, with 5 mice per group. Mice were sacrificed on day 38, after which spleens were harvested from each mouse.

### Isolation and *in vitro* restimulation of splenocytes

From each mouse, homogenized splenocytes were treated with erythrocyte lysis buffer (8.3 g/L NH_4_CL, 1 g/L NaHCO_3_, 5000 IE/L Heparin in dH_2_0; pH 7.4) and transferred to 24-well plates (6 × 10^6^ cells/well). The cells were cultured in IMDM medium (Gibco) supplemented with 8% FCS, 100 units penicillin, 100 units streptomycin, 2.92 mg/ml L-glutamine, and 20 μM β-mercaptoethanol (Sigma) at 37 °C in a humidified atmosphere of 5% CO_2_. The cells were either left unstimulated or stimulated for 24 hours with a combination of Prn, Ptx, and FHA (1 μg/ml each) (2 replicate wells per condition), after which the cells were harvested and pooled per culture condition per mouse.

### CD4^+^ T cell isolation and purity check

From each cultured splenocyte sample CD4^+^ T cells were isolated by positive selection using CD4 magnetic microbeads and a magnetic cell separator (Miltenyi Biotech) according to the manufacturer’s instructions. The purity of the CD4^+^ T cells was determined using flowcytometry. Briefly, the isolated cells were stained with Pacific blue-conjugated anti-CD4 (Biolegend) in FACS buffer (PBS (pH 7.2) supplemented with 0.5% BSA (Sigma Aldrich) and 0.5 mM EDTA (ICN Biomedicals). After washing, data were acquired on a FACS Canto II (BD Biosciences) and analyzed using FlowJo software (Tree Star). The purity of the isolated CD4^+^ T cells was >95%.

### RNA extraction

From each CD4^+^ T cell preparation, cells were lysed in Qiazol (Qiagen) and RNA isolation was performed using a miRNeasy Mini Kit with DNAse treatment (Qiagen) according to the manufacturer’s protocol. RNA concentrations and quality were determined using respectively UV spectroscopy (Tech3 module, Synergy Mx, BioTek) and electrophoresis (RNA nano 6000 kit, 2100 Bioanalyzer, Agilent Technologies).

### Microarray analysis

Amplification, labeling and hybridization of RNA samples to microarray chips (GeneChip HT MG-430 PM Array Plate; Affymetrix) were carried out at the Microarray Department of the University of Amsterdam (The Netherlands) according to Affymetrix protocols. Array plates were scanned with a Genechip HT array plate scanner and analyzed with the Affymetrix HT software suite. Microarray analysis was performed on 3 unstimulated and 5 antigen-stimulated samples per group.

### Data analysis of gene expression

Quality control and normalization of Affymetrix CEL files were performed using the ArrayAnalysis website (www.arrayanalysis.org)^57^, using the Robust Multichip Average (RMA) method^58^ and the MBNI custom CDF version 15^59^. Normalized data consisted of Log2 transformed signal values for 17306 genes. All slides passed quality control. Further analysis of normalized data was performed in R (www.r-project.org) and Microsoft Excel. Genes differentially expressed between the different groups of immunized mice were identified by using ANOVA. Fold ratio induction or repression of individual genes was calculated by comparing mean gene expression levels of the different immunization groups. Probes were considered differentially expressed if they met the following two criteria: (i) a p-value 0.001 (ANOVA), which corresponds to a Benjamini-Hochberg False discovery rate (FDR) of 5%; and (ii) an absolute fold ratio ≥1.5. Heatmaps visualizing differently expressed genes were made using GeneMaths XT software (Applied Maths). Hierarchical clustering of the differentially expressed genes was performed in GeneMaths XT software using Euclidean distance (with variances) as a distance metric and UPGMA linkage. Additional data visualization was done by Principal Component Analysis in R. Functional enrichment with an over-representation analysis (ORA) was performed using DAVID^17^ based on Gene Ontology biological processes (GO-BP) and Kyoto Encyclopedia of Genes and Genomes (KEGG) databases.

### Transcription factor-binding site analysis

For the transcription factor binding site (TFBS) analysis, the platform oPOSSUM3.0 (http://​opossum.​cisreg.​ca/​oPOSSUM3) was used. To evaluate whether a TFBS is enriched within the different gene sets, the software detects known transcription factor binding sites in the promoter sequences of the co-expressed genes^60^. Up- and downstream sequences (5000 bp) of up- or downregulated genes in CD4^+^ T cells of aP- or aP+LpxL1-vaccinated mice were analyzed using the default parameters in oPOSSUM 3.0 Single Site Analysis (SSA). A TFBS was considered enriched when it met the following criteria, Z-score >10 and Fischer score >7, which are the recommended criteria at the oPOSSUM site.

### Gene network analysis

To construct a gene-function network, genes associated with Th subsets and metabolism were determined using text mining in murine and human studies. Interactions between genes associated with Th subsets and metabolism were determined using the STRING database (http://string.embl.de/) with high confidence (0.700) and using co-occurrence, co-expression, experiments, databases, and text mining as types of evidence. The network visualization was performed using Cytoscape (version 2.8.3).

## Additional Information

**How to cite this article**: Brummelman, J. *et al.* Transcriptome signature for dampened Th2 dominance in acellular pertussis vaccine-induced CD4^+^ T cell responses through TLR4 ligation. *Sci. Rep.* **6**, 25064; doi: 10.1038/srep25064 (2016).

## Supplementary Material

Supplementary Information
