# Supplementary material for: Transcriptome signature for dampened Th2 dominance in acellular pertussis vaccine-induced CD4+ T cell responses through TLR4 ligation
Source: Sci Rep. 2016 Apr 27;6:25064. doi: 10.1038/srep25064 (PMC4846868; doi:10.1038/srep25064)
Supplement: Supplementary Information [file srep25064-s1.pdf]

Transcriptome signature for dampened Th2 dominance in acellular pertussis vaccine-induced CD4$^+$ T cell responses through TLR4 ligation

Jolanda Brummelman[1,4*], René H.M. Raeven[2], Kina Helm[1], Jeroen L.A. Pennings[3], Bernard Metz[2], Willem van Eden[4], Cécile A.C.M. van Els[1+], and Wanda G.H. Han[1+]

[1]*Centre for Infectious Disease Control, National Institute for Public Health and the Environment, Bilthoven, The Netherlands,* [2]*Intravacc, Institute for Translational Vaccinology, Bilthoven, The Netherlands,* [3]*Centre for Health Protection, National Institute for Public Health and the Environment, Bilthoven, The Netherlands,* [4]*Department of Infectious Diseases and Immunology, Utrecht University, The Netherlands,* [+]These authors contributed equally

Supplementary Information

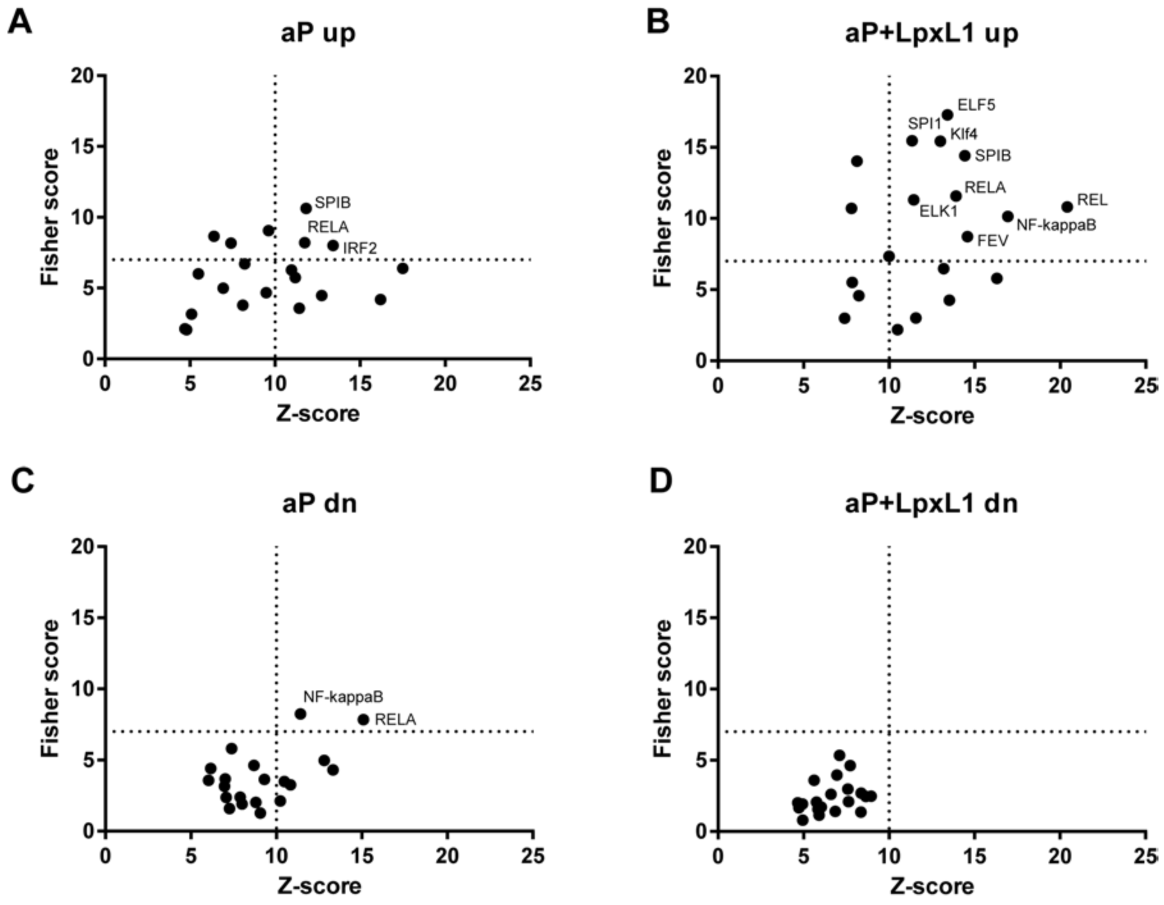

Supplementary Figure S1 – Over-representation of transcription factor-binding sites. Using the web-based platform oPOSSUM3.0 (http://opossum.cisreg.ca/oPOSSUM3) over-representation of transcription factor binding sites (TBFS) was analyzed within all upregulated genes in $CD4^+$ T cells of aP-vaccinated (A) or aP+LpxL1-vaccinated (B) mice, or within all downregulated genes in $CD4^+$ T cells of aP-vaccinated (C) or aP+LpxL1-vaccinated (D) mice. A TFBS was considered over-represented when it met the following criteria, Z-score>10 and Fischer score >7, which are the recommended criteria at the oPOSSUM site.

Supplementary Table S1 – Top 20 TFBS found in gene sets from CD4[+] T cells of aP- or aP+LpxL1-vaccinated mice.

Top 20 over-represented TFBS within upregulated genes in CD4[+] T cells of aP vaccinated mice (A)							
Transcription factor	JASPAR ID	Class	Family	Target gene hits	Target TFBS hits	Z-score[1]	Fisher score
REL	MA0101.1	Ig-fold	Rel	87	291	17.504	6.387
Pax4	MA0068.1	Helix-Turn-Helix	Homeo	3	3	16.198	4.191
IRF2	MA0051.1	Winged Helix-Turn-Helix	IRF	14	14	13.414	8.012
Stat3	MA0144.1	Ig-fold	Stat	79	227	12.747	4.469
SPIB	MA0081.1	Winged Helix-Turn-Helix	Ets	140	1449	11.833	10.628
RELA	MA0107.1	Ig-fold	Rel	65	131	11.748	8.211
FEV	MA0156.1	Winged Helix-Turn-Helix	Ets	115	693	11.428	3.568
NF-kappaB	MA0061.1	Ig-fold	Rel	69	172	11.185	5.745
ELK1	MA0028.1	Winged Helix-Turn-Helix	Ets	107	478	10.969	6.278
ELF5	MA0136.1	Winged Helix-Turn-Helix	Ets	133	959	9.624	9.058
STAT1	MA0137.2	Ig-fold	Stat	54	98	9.482	4.676
Pax6	MA0069.1	Helix-Turn-Helix	Homeo	17	17	8.211	6.71
Hand1::Tcfe2a	MA0092.1	Zipper-Type	Helix-Loop-Helix	105	477	8.111	3.784
MEF2A	MA0052.1	Other Alpha-Helix	MADS	64	121	7.405	8.167
EBF1	MA0154.1	Zipper-Type	Helix-Loop-Helix	91	324	6.954	4.987
SPI1	MA0080.2	Winged Helix-Turn-Helix	Ets	128	789	6.414	8.651
NFATC2	MA0152.1	Ig-fold	Rel	121	726	5.493	6.012
NR3C1	MA0113.1	Zinc-coordinating	Hormone-nuclear Receptor	22	28	5.094	3.164
EWSR1-FLI1	MA0149.1	Winged Helix-Turn-Helix	Ets	3	3	4.797	2.055
FOXF2	MA0030.1	Winged Helix-Turn-Helix	Forkhead	31	51	4.707	2.12

Top 20 over-represented TFBS within upregulated genes in CD4[+] T cells of aP+LpxL1 vaccinated mice (B)							
Transcription factor	JASPAR ID	Class	Family	Target gene hits	Target TFBS hits	Z-score[1]	Fisher score
REL	MA0101.1	Ig-fold	Rel	142	427	20.407	10.808
NF-kappaB	MA0061.1	Ig-fold	Rel	114	270	16.938	10.145
Stat3	MA0144.1	Ig-fold	Stat	125	341	16.304	5.797
FEV	MA0156.1	Winged Helix-Turn-Helix	Ets	192	1036	14.583	8.724
SPIB	MA0081.1	Winged Helix-Turn-Helix	Ets	222	2152	14.425	14.405
RELA	MA0107.1	Ig-fold	Rel	103	193	13.908	11.579
Hand1::Tcfe2a	MA0092.1	Zipper-Type	Helix-Loop-Helix	165	737	13.518	4.257
ELF5	MA0136.1	Winged Helix-Turn-Helix	Ets	217	1444	13.409	17.278
STAT1	MA0137.2	Ig-fold	Stat	86	150	13.184	6.474
Klf4	MA0039.2	Zinc-coordinating	BetaBetaAlpha-zinc finger	181	1006	12.993	15.421
Pax4	MA0068.1	Helix-Turn-Helix	Homeo	3	3	11.558	3.002
ELK1	MA0028.1	Winged Helix-Turn-Helix	Ets	175	695	11.443	11.31
SPI1	MA0080.2	Winged Helix-Turn-Helix	Ets	208	1215	11.341	15.465
TP53	MA0106.1	Zinc-coordinating	Loop-Sheet-Helix	1	1	10.49	2.186
EBF1	MA0154.1	Zipper-Type	Helix-Loop-Helix	146	491	9.99	7.34
IRF2	MA0051.1	Winged Helix-Turn-Helix	IRF	15	15	8.227	4.583
SP1	MA0079.2	Zinc-coordinating	BetaBetaAlpha-zinc finger	164	792	8.115	14.017
Pax6	MA0069.1	Helix-Turn-Helix	Homeo	22	23	7.843	5.516
MEF2A	MA0052.1	Other Alpha-Helix	MADS	100	175	7.795	10.702
EWSR1-FLI1	MA0149.1	Winged Helix-Turn-Helix	Ets	5	5	7.401	2.985

Top 20 over-represented TFBS within downregulated genes in CD4[+] T cells of aP vaccinated mice							
Transcription factor	JASPAR ID	Class	Family	Target gene hits	Target TFBS hits	Z-score[1]	Fisher score
RELA	MA0107.1	Ig-fold	Rel	82	160	15.089	7.836
CEBPA	MA0102.2	Zipper-Type	Leucine Zipper	132	512	13.306	4.313
IRF1	MA0050.1	Winged Helix-Turn-Helix	IRF	69	129	12.8	4.975
NF-kappaB	MA0061.1	Ig-fold	Rel	94	197	11.408	8.238
REL	MA0101.1	Ig-fold	Rel	104	298	10.831	3.265

Pou5f1	MA0142.1	Helix-Turn-Helix	Homeo	35	53	10.466	3.484
SRY	MA0084.1	Other Alpha-Helix	High Mobility Group	149	900	10.233	2.121
IRF2	MA0051.1	Winged Helix-Turn-Helix	IRF	12	13	9.305	3.641
Sox17	MA0078.1	Other Alpha-Helix	High Mobility Group	134	633	9.059	1.267
FOXI1	MA0042.1	Winged Helix-Turn-Helix	Forkhead	120	436	8.792	2.032
NFATC2	MA0152.1	Ig-fold	Rel	155	871	8.686	4.624
Pax4	MA0068.1	Helix-Turn-Helix	Homeo	2	2	7.997	1.911
MEF2A	MA0052.1	Other Alpha-Helix	MADS	67	140	7.877	2.385
AP1	MA0099.2	Zipper-Type	Leucine Zipper	168	1090	7.388	5.808
Foxq1	MA0040.1	Winged Helix-Turn-Helix	Forkhead	77	190	7.258	1.596
TAL1::TCF3	MA0091.1	Zipper-Type	Helix-Loop-Helix	72	138	7.064	2.383
CTCF	MA0139.1	Zinc-coordinating	BetaBetaAlpha-zinc finger	34	40	7.008	3.675
HLF	MA0043.1	Zipper-Type	Leucine Zipper	57	93	6.971	3.164
NFE2L2	MA0150.1	Zipper-Type	Leucine Zipper	64	98	6.156	4.404
FEV	MA0156.1	Winged Helix-Turn-Helix	Ets	151	746	6.033	3.577

Top 20 over-represented TFBS within downregulated genes in CD4+ T cells of aP+LpxL1 vaccinated mice

Transcription factor	JASPAR ID	Class	Family	Target gene hits	Target TFBS hits	Z-score[1]	Fisher score
CEBPA	MA0102.2	Zipper-Type	Leucine Zipper	55	211	8.947	2.472
HNF4A	MA0114.1	Zinc-coordinating	Hormone-nuclear Receptor	30	57	8.647	2.461
NR3C1	MA0113.1	Zinc-coordinating	Hormone-nuclear Receptor	13	17	8.374	2.699
IRF1	MA0050.1	Winged Helix-Turn-Helix	IRF	25	53	8.352	1.359
CTCF	MA0139.1	Zinc-coordinating	BetaBetaAlpha-zinc finger	18	19	7.731	4.622
IRF2	MA0051.1	Winged Helix-Turn-Helix	IRF	5	6	7.628	2.094
RELA	MA0107.1	Ig-fold	Rel	32	61	7.584	2.969
NF-kappaB	MA0061.1	Ig-fold	Rel	41	80	7.105	5.341
Hand1::Tcfe2a	MA0092.1	Zipper-Type	Helix-Loop-Helix	61	232	6.954	3.95
Pax4	MA0068.1	Helix-Turn-Helix	Homeo	1	1	6.848	1.405
ELF5	MA0136.1	Winged Helix-Turn-Helix	Ets	69	454	6.602	2.604
Foxq1	MA0040.1	Winged Helix-Turn-Helix	Forkhead	34	81	6.029	1.712
AP1	MA0099.2	Zipper-Type	Leucine Zipper	65	454	5.905	1.136
TAL1::TCF3	MA0091.1	Zipper-Type	Helix-Loop-Helix	30	59	5.843	1.561
SPI1	MA0080.2	Winged Helix-Turn-Helix	Ets	65	383	5.763	2.058
SPIB	MA0081.1	Winged Helix-Turn-Helix	Ets	74	663	5.619	3.587
MEF2A	MA0052.1	Other Alpha-Helix	MADS	25	57	4.946	0.794
EBF1	MA0154.1	Zipper-Type	Helix-Loop-Helix	47	154	4.936	1.926
znf143	MA0088.1	Zinc-coordinating	BetaBetaAlpha-zinc finger	7	8	4.731	1.652
Egr1	MA0162.1	Zinc-coordinating	BetaBetaAlpha-zinc finger	25	46	4.661	2.009

[1] Top 20 TFBS found in the different gene sets are arranged by Z-score
Significant results are shaded in blue.
